# miRmap: Comprehensive prediction of microRNA target repression strength

**DOI:** 10.1093/nar/gks901

**Published:** 2012-10-02

**Authors:** Charles E. Vejnar, Evgeny M. Zdobnov

**Affiliations:** ^1^Department of Genetic Medicine and Development, University of Geneva, Rue Michel-Servet 1, 1211 Geneva 4, ^2^Swiss Institute of Bioinformatics, 1211 Geneva, Switzerland and ^3^Imperial College London, South Kensington Campus, London, SW7 2AZ, UK

## Abstract

MicroRNAs, or miRNAs, post-transcriptionally repress the expression of protein-coding genes. The human genome encodes over 1000 miRNA genes that collectively target the majority of messenger RNAs (mRNAs). Base pairing of the so-called miRNA ‘seed’ region with mRNAs identifies many thousands of putative targets. Evaluating the strength of the resulting mRNA repression remains challenging, but is essential for a biologically informative ranking of potential miRNA targets. To address these challenges, predictors may use thermodynamic, evolutionary, probabilistic or sequence-based features. We developed an open-source software library, miRmap, which for the first time comprehensively covers all four approaches using 11 predictor features, 3 of which are novel. This allowed us to examine feature correlations and to compare their predictive power in an unbiased way using high-throughput experimental data from immunopurification, transcriptomics, proteomics and polysome fractionation experiments. Overall, target site accessibility appears to be the most predictive feature. Our novel feature based on PhyloP, which evaluates the significance of negative selection, is the best performing predictor in the evolutionary category. We combined all the features into an integrated model that almost doubles the predictive power of TargetScan. miRmap is freely available from http://cegg.unige.ch/mirmap.

## INTRODUCTION

MicroRNAs (miRNAs) are short (∼22 nt) non-coding RNAs that guide the RNA-induced silencing complex (RISC) to post-transcriptionally repress the expression of protein-coding genes by binding to targeted messenger RNAs (mRNAs) (1–3). The detailed mechanism of this guidance is not yet resolved, but exact pairing between the so-called ‘seed’ region, positions from 2 to 7 (or 8) from the 5′-end of the miRNA, and the 3′-UTR of the mRNA is believed to be necessary for most animal miRNA–mRNA interactions (4). Such miRNA seed pairing with a 3′-UTR of an mRNA, however, is not always sufficient for a functional interaction (4), and in a few specific cases, non-canonical pairing (non-Watson–Crick pairing) with G:U wobbles or mismatches may be acceptable (4,5). Nevertheless, in all recent large-scale miRNA experiments (6–9), the strongest prediction signal remains the presence of seed matching sites in regulated mRNAs, and therefore, it is commonly used as a mandatory signal in functional assays. Since the seed match spans only six or seven nucleotides, many of such matches may occur simply by chance. Searching for longer seed matches, which are less likely to occur by chance but also yield stronger repression, therefore increases the specificity while reducing the sensitivity of the target search. Indeed, the seed definition has a prominent effect on the sensitivity (10). Even with a stringent seed definition, there are still many potential miRNA targets, and experimentally testing all miRNA–mRNA combinations having a seed match is practically not feasible. Prioritization of targets for any miRNA functional analysis is therefore of critical importance. This necessitates the ranking of potential miRNA targets bearing a seed, not only predicting in a binary manner if an mRNA is a target or not. A biologically meaningful ranking criterion is the miRNA-mediated repression strength that can be experimentally measured as the effect on mRNA or protein levels. We used a collection features to computationally predict the miRNA repression strength from additional information beyond the seed match, and thereby rank putative miRNA–mRNA interactions in a biologically relevant manner.

The interaction between a miRNA and its mRNA target site can be considered from (i) a thermodynamic, (ii) a probabilistic, (iii) an evolutionary or (iv) a sequence-based point of view. Several computational tools (11) for miRNA target site prediction have been developed that use one or more of these aspects (Table 1). This overlap hinders effective comparisons of individual predictor performances, which may use overlapping sets of prediction features and variable implementations of the same approaches. Moreover, most of these programs are not freely available, complicating direct comparisons (20). To avoid this type of benchmarking bias, more recent studies (21–23) have recomputed predictions with limited sets of features focusing on binary predictions of target or non-target instead of considering the strength of repression. Ignoring the fact that miRNA repression strength displays a continuous range of strong-to-weak effects makes the distinction between target and non-target a matter of choosing an arbitrary cutoff. Here, we present a thorough comparison of the power of individual approaches to predict the repression strength of miRNA–mRNA pairs, assessed using data from transcriptomics, immunopurification (IP), proteomics and polysome fractionation high-throughput experiments. This was achieved using miRmap, our implementation of a comprehensive set of prediction features that we have made available as an open-source Python library. The features encompass the thermodynamic, conservation, probabilistic and sequence-based categories; eight features have been described previously in the literature, while three are novel features, each from a different category. We examined correlations among the features, measured the predictive power and combined all of them into an integrated prediction model.

**Table 1.** Approaches used by miRNA target prediction software tools

	Thermodynamic	Evolutionary	Probabilistic	Sequence-based	References
miRmap	✓	✓	✓	✓	
TargetScan		✓^a^		✓	Grimson *et al.* (6)
PITA	✓				Kertesz *et al.* (12)
PicTar	✓	✓	✓		Krek *et al.* (13)
miRanda	✓				John *et al.* (14)
RNAhybrid	✓				Rehmsmeier *et al.* (15)
DIANA-microT	✓	✓			Kiriakidou *et al.* (16)
ElMMo		✓	✓		Gaidatzis *et al.* (17)
PACMIT	✓		✓		Marín and Vanícek (18)

^a^We used the TargetScan context score (6). An evolutionary approach was latter added in TargetScan (19), but it is a separated filter not included in the context score model.

## MATERIALS AND METHODS

### Experimental data

Expression microarrays of miRNA-transfected HeLa cells from Grimson *et al.* (6) were downloaded from GEO (GSE8501) for miRNAs 122 a, 128 a, 132, 133 a, 142, 148 b, 181 a, 7 and 9. We used expression data at 24 h post-transfection and, similar to Grimson *et al.* (6), only selected probes with signal intensities above the median in the control transfection experiments to retain only the transcripts expressed enough to observe miRNA silencing.

Similarly, we downloaded the expression microarrays from Linsley *et al.* (24) from GEO (GSE6838). We used the experiments GSM156522, GSM156523, GSM156524, GSM156545, GSM156546, GSM156547, GSM156548, GSM156553, GSM156557, GSM156559, GSM156576, GSM156577, GSM156578, GSM156579 and GSM156581, measured at 24 h with the same experimental conditions. We applied the same selection filter as above (6).

We downloaded the Selbach *et al.* (7) proteomics over-expression data directly from the web site dedicated to the article. We included expression fold-changes measured at 32 h for miR-1, miR-155 and miR-16 but excluded let-7 b and miR-30 a as these miRNAs exert a negative feedback effect on the RNA silencing pathway (7,21).

HITS-CLIP data from the Chi *et al.* (9) study were also downloaded from the web site dedicated to the article. After cross-linking Argonaute (Ago) with its neighbouring RNAs, the authors immunopurified Ago and sequenced the pulled-down RNAs. We used the peak height as a measure of miRNA targeting for the 20 available most abundant miRNAs and filtered the relevance of the peaks using a biological complexity (BC, a measure of reproducibility between biological replicates) criterion strictly superior to 1 for medium stringency.

Hendrickson *et al.* (25) injected miR-124 into HEK293T cells and measured (i) the miR-RISC association with Ago IP, (ii) transcriptome expression with microarrays and (iii) translation activity with polysome fractionation. We used dataset number 5 from the Supplementary Information which includes all measurements for each transcript.

### Sequence data

RefSeq 47 (26) mapped on the human (hg19) and mouse (mm9) genomes by the UCSC (27) were used to define mRNA annotations, restricted to ‘NM_’ transcripts. miRBase 16 (28) was used for miRNA annotations.

### Target prediction features

#### Thermodynamics of miRNA–mRNA interactions

The miRNA–mRNA pair forms an RNA duplex. Using the Vienna RNA Secondary Structure library (29), we computed the minimum free folding energy (MFE) of this duplex (with the ‘cofold’ function), and named it ‘ΔG duplex’. While the structure with the lowest predicted energy or MFE is the most stable structure, populations of RNAs adopt different sub-optimal structures *in vivo*. We computed the ensemble free energy of the binding (with the ‘co_pf_fold’ function), and named this feature ‘ΔG binding’. We used the ‘cofold’ function for the ‘ΔG duplex’ computation as this function of the Vienna RNA Secondary Structure library is more appropriate than the modified ‘duplexfold’ function used in PITA (12) to compute this feature. The ‘duplexfold’ function was written to quickly scan for possible hybridization sites, whereas the ‘cofold’ function, albeit being more computationally intensive, was specifically designed to compute the duplex free energy taking into account intra-molecular and inter-molecular pairs.

The RISC is much larger than the miRNA (30) and must bind to the mRNA in an extended single-stranded form. We computed the energy required to unfold the 3′-UTR region of the target site (this area can be optionally extended), named ‘ΔG open’, similarly to PITA (12), with the ‘pf_fold’ function from the Vienna Library (29). The computation of ‘ΔG open’ requires two energy calculations; the free energy of the mRNA constrained to maintain the target site single stranded is subtracted from the free energy of the same unconstrained mRNA. The single-strand constraint was placed on a segment of 70 nucleotides centred on the target site. Finally, ‘ΔG open’ summed with ‘ΔG duplex’ or ‘ΔG binding’ gives the total system energy: we named it ‘ΔG total’ (named ΔΔG in PITA (12)).

#### Probability of the motif occurrence

We modelled the 3′-UTR sequence as a Markov process (order 1, as 3′-UTR sequences are too short to parameterize higher orders) and determined the expected probability of finding at least *n* occurrences of the motif defined as either an exact seed match or the full miRNA binding site, using two different methods. In the first method, the probability distribution was approximated with a binomial distribution, as in Marín and Vanícek (18), while in the second method, we computed the exact probability distribution based on the theoretical work of Nuel *et al.* (31).

#### Conservation of the target site

Using the UCSC (27) MultiZ multiple genome sequence alignments (hg19, MultiZ 46-way; mm9, MultiZ 30-way), we searched for conserved miRNA target sites in the alignment blocks defined by the 3′-UTRs of the reference species (human or mouse for the HITS-CLIP data). From a mammalian species tree (UCSC (27)), we first pruned all the species that did not contain the target site. We then summed the lengths of the remaining branches (as in (32)) to obtain the branch length score (BLS). As implemented by Friedman *et al.* (19), we summed the branch lengths of the species topology fitted for each 3′-UTR alignment with the REV model using the PhyloFit program from the PHAST suite (33). Tree manipulations were done with the DendroPy (34) library.

To test for evidence of negative selection acting on miRNA target sites, we used the Siepel, Pollard and Haussler (SPH) test implemented in the PhyloP program of the PHAST suite (33). This test evaluates if the branch lengths of the tree built from the target sites are significantly shorter (less divergent because of negative selection) than the background (the 3′-UTR as for the previous method). The reported values in the text are the test −log(*P*-value).

PhastCons 46-way run data from UCSC (27) were used to compute the average seed match probability to be a conserved element. The PhastCons scores of each base in the seed were averaged to obtain the seed score (23,35).

#### Sequence features

We implemented the three sequence features of the TargetScan context score (6): (i) the A and U nucleotide ratio over G and C, weighted around the seed match, (ii) the 3′-compensatory pairing feature and (iii) the distance between the target site and the nearest 3′-UTR end.

### Relative importance of features

We computed the relative importance of features in the multiple linear models with the CAR method (36) which decomposes the proportion of the variance explained by each variable of a model while taking the correlations among variables into account.

## RESULTS

### miRNA target prediction library

**Figure 1.**
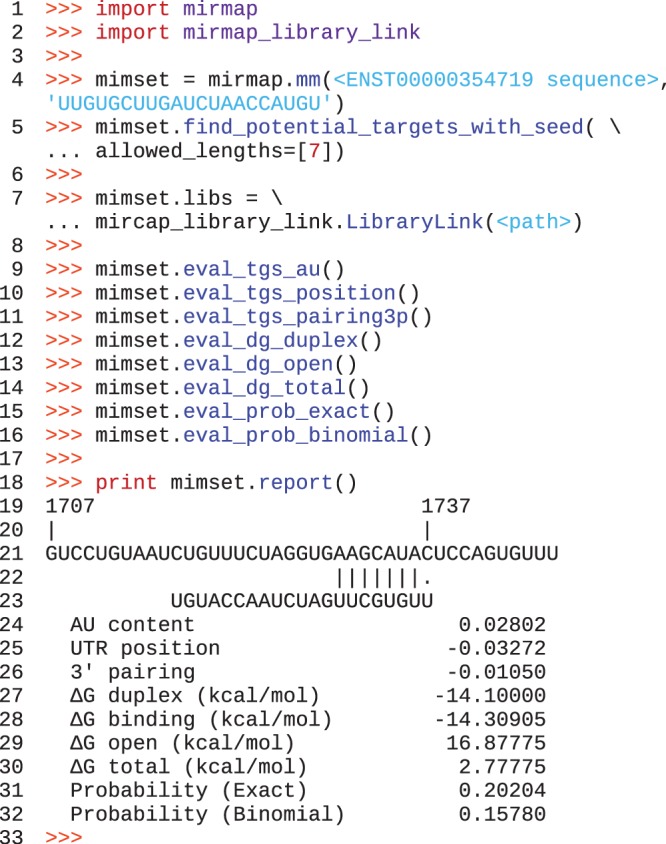
miRmap library usage: after importing the library (lines 1 and 2), a ‘mimset’ object is created containing the mRNA and miRNA sequences. We then call a method of the mimset object to search (line 5) for seeds with a length of 7 (all parameters have defaults that can be changed this way). The link with the C libraries is initalized on line 7. We then manually evaluate the repression strength with differents methods (lines 9–16). Each of these methods have modifiable parameters. We finally print a report (line 18).

We developed a comprehensive prediction model implemented as the miRmap open-source Python library (Figure 1) with a total of 11 features covering a wide range of published and novel methods (Table 2). With our own implementation, we compared the different features without the biases inherent to comparison of pre-computed predictions. We evaluated the features' individual predictive power, measured their intercorrelations and examined different combinations of methods. Additionally, in order to facilitate the library usage, five features are implemented in pure Python.

**Table 2.** miRNA target prediction features of the miRmap library

Category	Feature	Description	Python-only	Remarks
Thermodynamic	ΔG duplex	MFE with RNAcofold		
	ΔG binding	Binding energy based on ensemble free energy		*New feature*
	ΔG open	mRNA opening free energy—Accessibility		As in PITA (12)
	ΔG total	ΔG Duplex + ΔG open		Similar to ΔΔG in PITA (12)
Probabilistic	P.over binomial	Site over-representation prob. (binomial dist.)	✓	As in PACMIT (18)
	P.over exact	Site over-representation prob. (exact dist.)		*New feature*
Conservation	BLS	Branch length score on 3′-UTR fitted tree	✓	Similar to Stark *et al.* (32)
	PhyloP	SPH test from PhyloP		*New feature*
Sequence	AU content	AU nucleotide composition around the seed	✓	As in TargetScan (6)
	UTR position	Distance from the nearest 3′-UTR end	✓	As in TargetScan (6)
	3′-pairing	3′-compensatory pairing	✓	As in TargetScan (6)

Novel methods include (i) a more accurate way to compute the binding energy between the miRNA and the mRNA based on the ensemble free energy instead of the minimum free energy, (ii) an exact method to compute the probability that the seed match is an over-represented motif in the 3′-UTR and (iii) a non-empirical statistical test to assess the significance of target site evolutionary conservation.

#### ΔG binding

miRNAs bind to their targeted mRNAs forming a helix. The minimum free folding energy (MFE) of these duplexes can be computed (‘ΔG duplex’) but the structure with the MFE only represents a fraction of the possible and existing structures. Additionally, ‘ΔG duplex’ is a measure of the energy of the entire double-stranded structure, it does not describe the binding energy itself. This is captured by the ‘ΔG binding’ measurement, which represents only the binding energy computed from the ensemble free energy.

#### P exact

Within 3′-UTRs, only certain sequence regions have regulatory or structural roles. These regions can therefore be considered as islands of natural selection in a sea of mostly neutrally evolving sequence; ∼5% of the human 3′-UTR bases are constrained (37). This distinction can be exploited within a probabilistic (or evolutionary, see next paragraph) framework to distinguish the background sequence composition from the target site composition. Having modelled the background sequence composition (with a Markov process, see ‘Materials and Methods’ section), it is possible to compute a probability distribution of motif occurrences in order to assess the significance of the site presence. Several approximations (e.g. Gaussian, Poisson, binomial or large deviation) can be used to compute the probability distribution depending on the sequence length and the expected number of motif occurrences. As 3′-UTR sequences are relatively short, we computed not only an approximate distribution (‘P.over binomial’) but also an exact distribution (‘P.over exact’).

#### PhyloP

Empirical distributions described previously (19,32) can be used to assess the statistical significance of the ‘BLS’ (see ‘Materials and Methods’ section). Alternatively, a theoretical framework (33) may be used to test for significant natural selection; the SPH test evaluates the probability that part of a sequence is under selection, in our case negative selection. This framework relies on a comparison of the reference tree built from the complete 3′-UTR multiple sequence alignment and the tree built from the target site (the sequence region delineated by the seed match or the full target site) multiple sequence alignment.

For a meaningful comparison of a potential target site to the complete 3′-UTR, each of the sequences in the target site alignment should be a recognizable miRNA binding site. In other words, for the ‘PhyloP’ feature to produce meaningful results, target site positions should be conserved among species. To test this condition, potential target sites were identified by searching the 3′-UTR alignments of all human mRNAs for matches to all known human miRNA seeds. Positions are conserved for the majority of human seed matches; on average, 76% of the human seed matches are found at the same position in the alignment for the other mammalian species. For this analysis, sequences of species in the alignment without any seed match were discarded. According to this analysis, the turn-over of miRNA target sites in mammals seems to be low. The conservation of target site positions in the alignment supports our usage of PhyloP. Moreover, the percentages vary from 47 to 99% if we analyse each miRNA individually. The miRNAs with low complexity sequences tend to have low percentages, which also support the choice of this test as low complexity miRNAs have less specific target sites.

### Correlation among features

**Figure 2.**
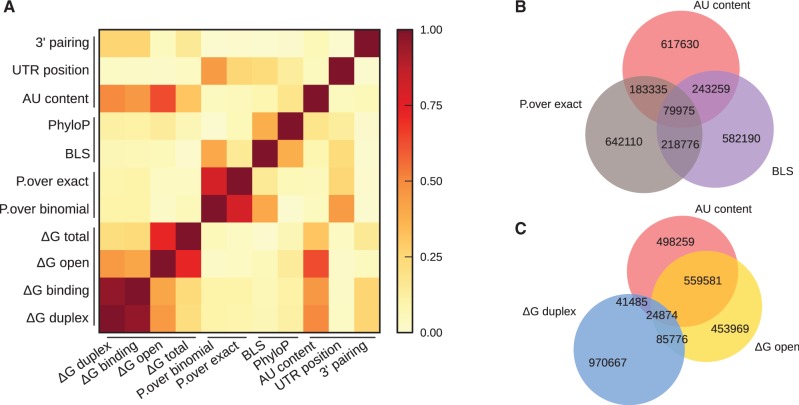
Correlation among features based on prediction for human miRNAs and mRNAs. (**A**) A heatmap of the absolute values of Spearman correlation coefficients between pairs of features classified in methods categories. Venn diagrams (**B**) and (**C**) show the overlaps among the first best prediction quartiles of selected features. One feature per category (sequence-based with ‘AU content’, conservation with the ‘BLS’ and probabilistic with ‘P.over exact’) is shown on (A). Venn diagram (C) underlines the high overlap between ‘AU content’ and ‘ΔG open’ that we grouped in the ‘accessibility group’, whereas ‘ΔG duplex’ has a very low overlap with these two features. We grouped ‘ΔG duplex’ with ‘ΔG binding’ in the ‘binding energy’ group. Numbers of predicted relationships between human miRNA and mRNA are written in the corresponding overlaps of the Venn diagrams.

We identified potential miRNA target sites by searching for matches to canonical 7-mer seeds on all 3′-UTRs of the human transcripts and predicted their strengths using the 11 methods of our miRmap library (see above and ‘Materials and Methods’ section). We focused our analysis on 7-mer seeds rather than shorter 6-mer seeds as stronger mRNA repression is associated with longer seeds. While this choice results in greater confidence in our feature performance analysis, target prediction with increased sensitivity could be easily obtained by integrating shorter seeds (see below). To evaluate the target site rankings computed with each feature and ignore other differences, e.g. their variances, we computed the Spearman rank correlation between feature pairs (Supplementary Table S1). The absolute values are plotted in Figure 2A.

The three most highly correlated feature pairs are those that measure the same underlying parameters using slightly different approaches: ‘ΔG duplex’ and ‘ΔG binding’ with 0.962, ‘P.over exact’ and ‘P.over binomial’ with 0.806 and ‘ΔG open’ and ‘ΔG total’ with 0.725. ‘ΔG open’ and ‘AU content’ show a correlation of −0.635; as folding algorithms rely on pairing and stacking energies that are stronger for GC than AU pairs, AU-rich sequences form potentially less stable structures, which explains the inverse correlation between ‘ΔG open’ and ‘AU content’. Since these two features evaluate the accessibility of mRNA to miRNA repression, we grouped them in an ‘accessibility group’ together with ‘ΔG total’.

As only the top miRNA target predictions are often used in experimental studies, we measured the overlap among features for their best quartiles. On the first Venn diagram (Figure 2B), we present one feature per group (accessibility, conservation and probabilistic), revealing the low overlap among these methods. The second Venn diagram (Figure 2C) confirms that ‘ΔG open’ and ‘AU content’ features belong to the same accessibility group whereas ‘ΔG duplex’ is a distinct feature not related to the target accessibility. However, target prediction program comparisons (see ‘Introduction’ section) often include PITA (12) which combines both ‘ΔG open’ and ‘ΔG duplex’, making any conclusions made in these comparisons about individual feature performance inaccurate.

### Individual feature performance

We evaluated the performance of each feature using data from seven experiments coming from five studies (Table 3) that cover different aspects of miRNA repression and use different assay techniques. (i) Chi *et al.* (9) performed an Ago-RNA cross-linking experiment followed by IP and sequencing from which miRNA binding sites were assayed. (ii) Hendrickson *et al.* (25) performed an Ago-IP without cross-linking that we included to underline the effect of the cross-linking step. To measure the effect on mRNA levels, we used studies based on miRNA transfections followed by microarray measurements from (iii) Grimson *et al.* (6), (iv) Linsley *et al.* (24) and (v) Hendrickson *et al.* (25). To assess the effect of miRNA on translation, we took advantage of polysome fractionation experiments from (vi) Hendrickson *et al.* (25), and of proteomics experiments from (vii) Selbach *et al.* (7) based on the pSILAC technology to obtain the final translation output.

**Table 3.** Experimental studies used to evaluate miRNA target prediction features

Dataset name	Type	Publication
Trans.Grimson	Microarray	Grimson *et al.* (6)
Trans.Linsley	Microarray	Linsley *et al.* (24)
Prot.Selbach	pSILAC	Selbach *et al.* (7)
IPcross.Chi	HITS-CLIP	Chi *et al.* (9)
IP.Hendrickson	Immunopurification	Hendrickson *et al.* (25)
Trans.Hendrickson	Microarray	Hendrickson *et al.* (25)
RibN.Hendrickson	Polysome fractionation	Hendrickson *et al.* (25)

We identified potential miRNA target sites by searching for matches to canonical 7-mer seeds on the transcripts involved in each experiment and predicted their strength with the 11 methods implemented in our miRmap library, and an additional feature derived from the PhastCons UCSC track (see ‘Materials and Methods’ section) to facilitate comparisons with Wen *et al.* (23) results. We then evaluated the correlations between the measured and predicted miRNA repression strengths.

**Figure 3.**
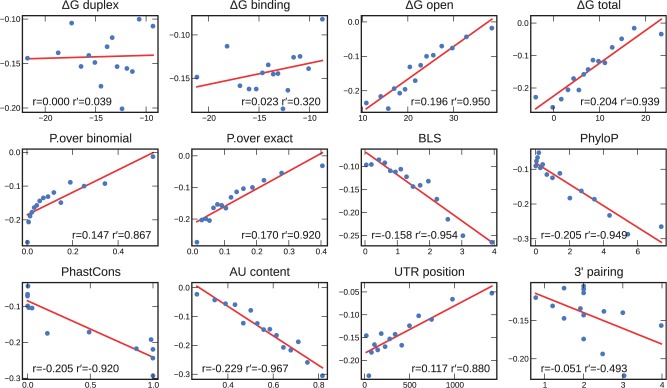
Correlation between each feature and the expression fold-changes of mRNAs following miRNA injection (‘Trans.Grimson’ dataset). Data points were binned in 15 equally sized bins. The average in each bin is represented by a blue dot. We fitted a linear regression model (red line) on the blue dots. *r* is the correlation on the full dataset; *r*′ is the correlation on the binned dataset. *P*-values can be found in Supplementary Table S2.

We focused our first analysis on the transcriptomics data, as these experiments measure a predominant effect of miRNA repression (38,39) and have the largest scale (‘Trans.Grimson’, ‘Trans.Linsley’ and ‘Trans.Hendrickson’ involve a total of 24 miRNAs). Figure 3 shows the linear regressions and correlations between each feature and the observed reductions in mRNA levels for the ‘Trans.Grimson’ dataset (Supplementary Table S2). The correlation coefficients range from 0.000 for the worst performing feature, ‘ΔG duplex’, to −0.229 for the best feature, ‘AU content’. The next best features are ‘PhyloP’, ‘PhastCons’, ‘ΔG total’, ‘ΔG open’, followed by ‘P.over exact’ and ‘BLS’. Two of our novel features show better correlations than their related features: (i) ‘PhyloP’ is the best performing conservation method (−0.205) and (ii) ‘P.over exact’ performs better than ‘P.over binomial’, i.e. computing the exact probability distribution is better than using the binomial approximation (0.170 versus 0.147). In addition, (iii) considering the ensemble energy outperforms using only the MFE (‘ΔG binding’: 0.023 versus ‘ΔG duplex’: 0.000).

**Figure 4.**
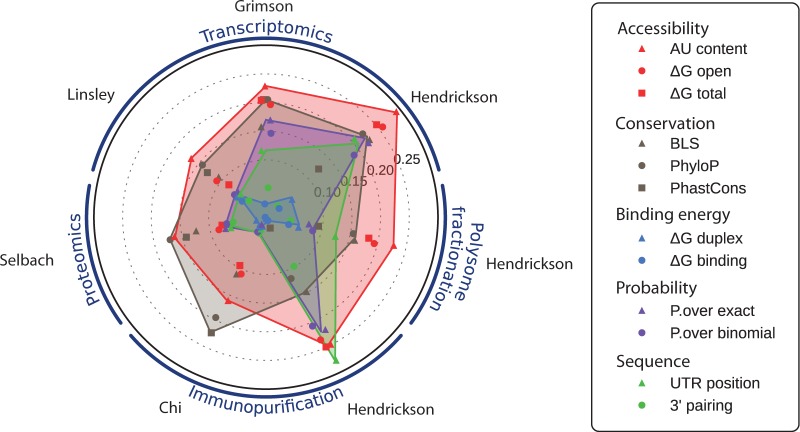
Correlation between each feature and the seven experimental miRNA repression measures (the name of the first author of each dataset is shown in grey) classified in transcriptomics, proteomics, IP and polysome fractionation experiment types. Target prediction features are organized into groups that aim to evaluate the same type of information. The radial axis represents the correlation coefficient (the highest correlations are the furthest from the centre of the circle).

In our second analysis, we examined all the datasets in order to compare the performance of each feature across additional aspects of miRNA repression, assessed through IP, proteomics and polysome fractionation experiments. Correlations for each feature and each experimental dataset are plotted in Figure 4 (Supplementary Table S2). Remarkably, feature performances show high consistency between each of the experimental datasets: accessibility features (red) always perform well, while binding energies (light blue) are always poorly predictive. As target sites in our study contain a seed, the part of the binding energy discriminating target sites is due to the seed nucleotide composition and to the pairing outside the seed. This energy does not drive the miRNA repression strength, as confirmed by the low performance of ‘3′-pairing’. Moreover, the ranking of each feature performance is very similar between datasets using the same experimental techniques, e.g. the ‘Trans.Grimson’ and ‘Trans.Linsley’ datasets. While based on only a single miRNA, the ‘Trans.Hendrickson’ dataset shows better overall performance with only minor differences: ‘UTR position’ improved its ranking while ‘PhastCons’ is outperformed by ‘BLS’.

‘AU content’ consistently provides the best measure of target site accessibility. This is in agreement with findings from Wen *et al.* (23), but in contrast to results from Hausser *et al.* (21), which described better performance with ‘ΔG open’ for an IP experiment. However, for the ‘IP.Hendrickson’ dataset, which, like Hausser *et al.* (21) involved IP without cross-linking, ‘AU content’ and ‘ΔG open’ perform equally well. The ‘IP.Hendrickson’ experiment is also distinguished by the probabilistic (purple) and ‘UTR position’ (green) features that outperform the conservation features (grey), which may be explained by the lower precision of this method (i.e. IP without cross-linking), performed with a single miRNA.

The best conservation feature performance is generally slightly lower than the best accessibility feature, but it outperforms ‘AU content’ for the proteomics and HITS-CLIP datasets. ‘PhastCons’ performance on the HITS-CLIP dataset is consistent with findings from Wen *et al.* (23). Our novel conservation feature, ‘PhyloP’, shows the best or tied-best performance for five out of the seven datasets. When outperformed, it is only marginally outperformed implying that ‘PhyloP’ is the best overall conservation feature.

Hendrickson *et al.* (25) polysome fractionation measured the miRNA effects as ribosome occupancy (fraction of a given gene’s transcripts associated with ribosomes) and ribosome density (the average number of ribosomes bound per unit length of coding sequence). Effects caused by the miRNA on both parameters were detected by the authors, but were substantially higher on the ribosome density, in agreement with the absence of correlation with the ribosome occupancy that we observed, i.e. this measurement is not quantitative. However, the ribosome density is a quantitative measure of the miRNA effect, as the correlations were as high or higher than those of the large-scale transcriptomics experiments. We observed again, as for all Hendrickson *et al.* (25) datasets, a higher correlation for the ‘UTR position’ feature, probably caused by the experimental setup.

### Combining prediction features

**Figure 5.**
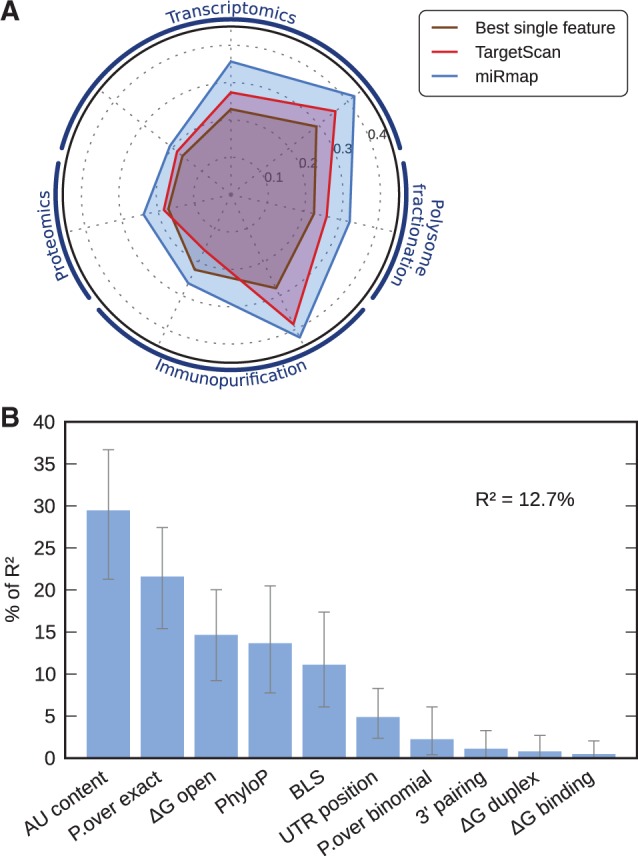
(**A**) Performance comparison (as coefficient correlations with experimental miRNA repression measures; order of the experiments is the same as Figure 4) of the best performing feature (brown), TargetScan context score (red) and miRmap (blue). (**B**) Feature relative importance in the miRmap multiple linear regression model predicting miRNA repression strength. *R*^2^ is the proportion of variance explained by the model. ‘AU content’ is the most explanatory variable with 29% of *R*^2^.

The features correlate linearly with experimentally measured miRNA repression levels. We combined 10 features of our miRmap library (we excluded ‘ΔG total’, as this feature is simply the sum of ‘ΔG duplex’ and ‘ΔG open’) with a multiple linear regression on the ‘Trans.Grimson’ dataset (*P* = 4.9 × 10^−^^110^; Supplementary Figure S7). This model explains 12.7% of the variance, close to a 2-fold increase over TargetScan context score (6): with the same type of regression, the three features of TargetScan context score (‘AU content’, ‘3′-pairing’ and ‘UTR position’) explain only 7.49% of the variance. This improved performance of our model is confirmed by the higher correlations with the experimental measurements, computed in the same manner as the individual feature correlations (Figure 5A). The contribution of each feature (on ‘Trans.Grimson’ dataset, Figure 5B) generally mirrors the rankings based on individual feature correlations: ‘AU content’ is the most explanatory feature, but ‘P.over exact’ contributes more in the regression model than its correlation rank suggests. Interestingly, the conservation features ‘PhyloP’ and ‘BLS’ contribute ∼14 and ∼11%, respectively, despite using the same input data (multiple genome sequence alignments) both contribute substantially to the explanation of the variance. Among the accessibility features, ‘ΔG open’ contributes only half as much as ‘AU content’ (15 and 30%, respectively). A model limited to the five features with the greatest contributions in the model with all the features (they represent 90.5% of the variance explanation of the full model) still explains 11.6% of the variance.

Instead of evaluating the model directly in terms of the explained variance, the quality of the ranking can be estimated by ordering the target sites by predicted strength, binning them and computing the mean expression fold-change of each bin. This approach, also used in (40) to evaluate the ranking of different tools for predicting miRNA repression strength on translation with proteomics data, was applied to 10 quantiles of the ordered predictions (Supplementary Figure S2). The overall distribution was shifted to lower fold-changes for miRmap compared with TargetScan context score, indicating a better ranking as a decrease in fold-change corresponds to greater repression. For the first quantile, the mean fold-change was reduced from −0.32 to −0.39 with miRmap.

Multiple linear regressions with the other datasets further support the conclusions from the analyses of individual feature performance (Supplementary Figures S1 and S3). They confirmed (i) the importance of ‘PhyloP’ for the ‘IPcross.Chi’ dataset (64% of *R*^2^) over 24% for ‘AU content’, (ii) the similar importance of ‘PhyloP’ and ‘AU content’ for proteomics (31% and 39% of *R*^2^, respectively) and (iii) the relevance of polysome fractionation experiment (‘RibN.Hendrickson’ dataset) to measure miRNA repression strength compared with proteomics as 10.6% of the variance was explained by the model (5.75% for proteomics). We also observed that the model computed on the ‘Trans.Linsley’ dataset explains only 4.36% of the variance even though this dataset is larger and based on the same techniques as the ‘Trans.Grimson’ dataset (*R*^2^ = 12.7%).

Shorter seeds may also promote miRNA repression, but usually with lower efficiencies (4). We therefore tested our approach on canonical 6-mer seeds by computing a model with these seed matches on the ‘Trans.Grimson’ dataset. While the global importance of each feature remained generally similar, with accessibility features being the most explanatory, *R*^2^ dropped to 8.31% of the variance (Supplementary Figure S4A), which still outperforms TargetScan context score (*R*^2^ = 4.70%). Interestingly, the importance of the ‘P.over exact’ probabilistic feature was reduced from 22 to 7%—falling from second position to fifth—as expected with shorter seeds where matches occur more frequently by chance and are therefore less statistically distinguishable from the background. We also evaluated the model by computing the distribution of fold-changes (Supplementary Figure S4B). As expected, the mean fold-changes were not as low as with the 7-mer seeds, nevertheless they confirmed the better ranking achieved with miRmap compared with TargetScan context score, e.g. the mean fold-change of the first quantile was reduced from −0.16 to −0.21. These results were further supported by the analysis of the other datasets (Supplementary Figures S5 and S6).

### Combining multiple target sites

Each mRNA can contain many miRNA target sites. Although most experimental datasets focus on a single miRNA at a time (or all miRNAs for the ‘IPcross.Chi’ dataset), a framework that can capture the multiplicity of these interactions should improve the predictive power. We examined three simple functions to combine the individual scores of target sites into a global metric at the mRNA level: the best (minimum or maximum depending on the sign of the correlation), the sum and the log of the sum of the exponentials. For this analysis, we selected transcripts from the ‘Trans.Grimson’ dataset with exactly two target sites, resulting in a sample size of 370 mRNAs (only 53 mRNAs have exactly three target sites). For this study, only features predicting different strengths for each target site in a 3′-UTR are appropriate as they would show different correlations for each function, thereby allowing function comparison. As the probabilistic features compute the probability of a fixed number of seed matches in the 3′-UTR, and as the ‘BLS’ score is also computed for the entire 3′-UTR, they could not be used.

The log of the sum of the exponentials function is designed to approximate interaction kinetics on the principle that stronger sites would drive the observed repression at the mRNA level. However, this function performed poorly for every feature as opposed to the sum (Supplementary Figure S8), which means that every target site has the same importance, indicating that the quantity of miRNA molecules is not limiting the repression reaction in this experiment. Regarding the binding energy features, ‘ΔG duplex’ and ‘ΔG binding’, the minimum energies provided the best predictors, i.e. the best site drives the repression for these two features. In contrast to their relatively poor performance with single site predictions, their performance was substantially increased (with correlations from 0 to 0.094 (*P* = 0.072) and 0.023 to 0.119 (*P* = 0.022) for ‘ΔG duplex’ and ‘ΔG binding’, respectively) but they still did not outperform the other features. The performance ranking among the remaining features was not substantially different to the single site predictions and, as already observed before (7), summing was the best option for the majority of them.

## DISCUSSION

We examined the performance of 12 features designed to predict the strength of miRNA repression on targeted mRNAs independently, and combined them into a linear model. This approach allowed us to assess feature accuracy to rank miRNA targets and avoid the choice of a threshold or the definition of a negative dataset (see ‘Introduction’ section). Overall, our combined features predict the strength of miRNA target repression more accurately: on the ‘Trans.Grimson’ dataset, our model explains 12.7% of the variance whereas TargetScan context score (‘AU content’, ‘3′-pairing’ and ‘UTR position’) explains 7.49% with the same type of linear model. We tested a more elaborate method than linear regression, the ensemble rule fitting, but it did not improve the predictions (data not shown). In our linear model, the feature explaining the largest part of the variance is the ‘AU content’ (29% of *R*^2^) which measures the accessibility of the miRNA target sites to the RISC. This result is consistent with TargetScan, but the proportion of the variance explained by this feature decreased from 74% of *R*^2^ in TargetScan to 29% in our model, as we included an additional method to compute the accessibility (‘ΔG open’). Indeed, the correlations among the features, and their individual performance across different datasets, revealed five distinct groups of prediction features. In particular, the accessibility group includes the thermodynamic evaluation of the cost to open the target site and the neighbouring structures (‘ΔG open’), and the ‘AU content’ feature, which are well correlated and performed similarly across all experimental datasets. Interestingly, ‘ΔG open’ is outperformed by ‘AU content’: computing a weighted partial (the stacking energy is ignored in the ‘AU content’ feature) accessibility feature is better than the allegedly more accurate feature that attempts to compute the ‘true’ accessibility.

Other miRNA target prediction tools (Table 1) consider a single or subset of our features. For example, PITA (13) considers only ‘ΔG total’, and PACMIT (18) a combination of ‘ΔG open’ and ‘P.over binomial’. As the performance of each of these features is lower than the combined approach of miRmap (Figure 4), these tools have less predictive power. While assessment of tools with different seed lengths, features and annotation sets have its caveats (see ‘Introduction’ section), TargetScan context score was the best performing tool according to large-scale proteomics experiments (7,8). As miRmap’s ranking of miRNA targets outperforms that of the TargetScan context score, we can speculate that our approach is the most predictive. Although we concentrated on 7-mer seeds, we showed that the same approach can be applied to 6-mer seeds, and it may also be used for the rarer centred seeds (41) to increase the overall prediction sensitivity (10).

The natural selection measured by either the ‘BLS’ or our ‘PhyloP’ feature is remarkably well correlated with the strength of repression: selected target sites are also sites of stronger repression. It is also known that older miRNAs have higher expression levels (42). Natural selection is acting on both the miRNA expression level and the repression strength to maximize the repression eﬀiciency. Furthermore, a correlation between the mRNA accessibility and the target site conservation has been shown in *Drosophila* (43) which can partially explain the good performance of the accessibility features (‘ΔG open’ and ‘AU content’) as this parameter is naturally selected. This dependence among the features partially explains why their individual performance is not additive in the global model. The probabilistic features also correlate with the conservation features but they are usually outperformed by the conservation features, even if they sometimes have similar performance (e.g. the probabilistic features are similar to the ‘BLS’ performance for the ‘Trans.Grimson’ dataset). In terms of computation, and more importantly of input data (multiple alignments, etc.), the probabilistic features are undoubtedly less expensive than the conservation features. They can therefore be seen as an alternative to an evolutionary approach, especially for organisms with long 3′-UTRs [between *Drosophila* and human their accuracy significantly drops in *Drosophila* (18)].

While we observed generally consistent results among the transcriptomics, polysome fractionation and proteomics experimental methods, they were distinguishable from IP experiments. The experimental methods measuring the repression, i.e. the effect of the miRNA, are more accurate to measure the repression strength than methods measuring only miRNA binding. Chi *et al.* (9) observed that 86% of conserved miR-124 seeds were present within the Ago footprint region, i.e. the HITS-CLIP method accurately identifies miRNA target sites but does not provide a quantitative measure of miRNA repression. We also noticed that, although polysome fractionation is not commonly used to test miRNA targets, the ribosome number measure performs as well as most other methods.

According to our model, a large part of the variance of the miRNA repression observed from experimental measurements remains to be explained. Indeed, the overall variance includes miRNA indirect effects, such as regulation feedback loops. The proportions of variance explained by our model or TargetScan are therefore underestimates of the explainable variance by miRNA direct repression. An improved understanding of the molecular mechanisms of repression, beyond the currently considered thermodynamic, evolutionary, probabilistic or sequence-based aspects will undoubtedly lead to better predictions. Nevertheless, our model shows that capturing more information with complementary features already significantly improves the predictive power. Additional considerations may extend these improvements. For example, our ‘PhyloP’ feature is based on a ‘per base’ model, i.e. positions in the alignment are considered independently. However, for RNAs in general, stacking energies are important, so a context-dependent model, when integrated in PHAST (33), should increase performance and would also quantify the importance of stacking energies. Other considerations are, for the moment, less tractable, e.g. taking the kinetics of the repression into account. The availability of the different components, such as enzymes, miRNAs and mRNAs is ignored in the existing models. However, this system approach requires substantially more information, notably the concentration of different components.

The miRmap library implements 11 features from 4 categories, making it currently the most comprehensive miRNA target prediction resource. All the features and the model evaluated in this study are available as an open-source Python library on a public revision control service, allowing tracking of all contributions. As such, miRmap establishes a solid foundation for the future development of approaches to miRNA target prediction, facilitating meaningful comparisons between existing and new features, and providing the community with direct access to state-of-the-art analytical tools.

## SUPPLEMENTARY DATA

Supplementary Data are available at NAR Online: Supplementary Tables 1 and 2 and Supplementary Figures 1–8.

## FUNDING

Swiss National Science Foundation [PDFMA3-118375 and 31003A-125350]. Funding for open access charge: Swiss Institute of Bioinformatics.

*Conflict of interest statement*. None declared.

## Supplementary Material

Supplementary Data
