# Supplementary material for: miRmap: Comprehensive prediction of microRNA target repression strength
Source: Nucleic Acids Res. 2012 Oct 2;40(22):11673–83. doi: 10.1093/nar/gks901 (PMC3526310; doi:10.1093/nar/gks901)
Supplement: Supplementary Data [file supp_40_22_11673__index.html]

miRmap: Comprehensive prediction of microRNA target repression strength — miRmap: Comprehensive prediction of microRNA target repression strength — Supplementary Data 

# miRmap: Comprehensive prediction of microRNA target repression strength

## Supplementary Data

files

**Files in this Data Supplement:**

- Supplementary Data - pdf file
