# Supplementary material for: miRmap: Comprehensive prediction of microRNA target repression strength
Source: Nucleic Acids Res. 2012 Oct 2;40(22):11673–83. doi: 10.1093/nar/gks901 (PMC3526310; doi:10.1093/nar/gks901)
Supplement: Supplementary Data [file supp_gks901_nar-01067-n-2012-File010.pdf]

miRmap: Comprehensive prediction of microRNA target repression strength

Supplementary information

The source code and documentation of the miRmap Python library is available online at:

http://cegg.unige.ch/mirmap

		ΔG duplex	ΔG binding	ΔG open	ΔG total	P.over binomial	P.over exact	BLS	PhyloP	AU content	UTR position	3′ pairing
ΔG duplex	r	1.000	0.962	-0.456	0.222	0.101	0.097	0.069	-0.118	0.507	0.029	-0.263
	p	0.00e+00	0.00e+00	0.00e+00	0.00e+00	0.00e+00	0.00e+00	0.00e+00	0.00e+00	0.00e+00	0.00e+00	0.00e+00
ΔG binding	r	0.962	1.000	-0.416	0.241	0.102	0.098	0.065	-0.110	0.464	0.026	-0.260
	p	0.00e+00	0.00e+00	0.00e+00	0.00e+00	0.00e+00	0.00e+00	0.00e+00	0.00e+00	0.00e+00	0.00e+00	0.00e+00
ΔG open	r	-0.456	-0.416	1.000	0.725	-0.024	-0.037	-0.058	0.142	-0.635	0.019	0.041
	p	0.00e+00	0.00e+00	0.00e+00	0.00e+00	0.00e+00	0.00e+00	0.00e+00	0.00e+00	0.00e+00	0.00e+00	0.00e+00
ΔG total	r	0.222	0.241	0.725	1.000	0.058	0.037	-0.005	0.070	-0.317	0.034	-0.157
	p	0.00e+00	0.00e+00	0.00e+00	0.00e+00	0.00e+00	0.00e+00	2.78e-27	0.00e+00	0.00e+00	0.00e+00	0.00e+00
P.over binomial	r	0.101	0.102	-0.024	0.058	1.000	0.806	0.405	-0.002	0.044	0.451	-0.003
	p	0.00e+00	0.00e+00	0.00e+00	0.00e+00	0.00e+00	0.00e+00	0.00e+00	1.05e-06	0.00e+00	0.00e+00	4.73e-11
P.over exact	r	0.097	0.098	-0.037	0.037	0.806	1.000	0.151	-0.060	0.051	0.256	-0.005
	p	0.00e+00	0.00e+00	0.00e+00	0.00e+00	0.00e+00	0.00e+00	0.00e+00	0.00e+00	0.00e+00	0.00e+00	9.41e-27
BLS	r	0.069	0.065	-0.058	-0.005	0.405	0.151	1.000	-0.386	0.079	0.232	-0.001
	p	0.00e+00	0.00e+00	0.00e+00	2.78e-27	0.00e+00	0.00e+00	0.00e+00	0.00e+00	0.00e+00	0.00e+00	1.99e-02
PhyloP	r	-0.118	-0.110	0.142	0.070	-0.002	-0.060	-0.386	1.000	-0.186	0.138	0.011
	p	0.00e+00	0.00e+00	0.00e+00	0.00e+00	1.05e-06	0.00e+00	0.00e+00	0.00e+00	0.00e+00	0.00e+00	7.86e-114
AU content	r	0.507	0.464	-0.635	-0.317	0.044	0.051	0.079	-0.186	1.000	0.044	-0.062
	p	0.00e+00	0.00e+00	0.00e+00	0.00e+00	0.00e+00	0.00e+00	0.00e+00	0.00e+00	0.00e+00	0.00e+00	0.00e+00
UTR position	r	0.029	0.026	0.019	0.034	0.451	0.256	0.232	0.138	0.044	1.000	-0.000
	p	0.00e+00	0.00e+00	0.00e+00	0.00e+00	0.00e+00	0.00e+00	0.00e+00	0.00e+00	0.00e+00	0.00e+00	7.35e-01
3′ pairing	r	-0.263	-0.260	0.041	-0.157	-0.003	-0.005	-0.001	0.011	-0.062	-0.000	1.000
	p	0.00e+00	0.00e+00	0.00e+00	0.00e+00	4.73e-11	9.41e-27	1.99e-02	7.86e-114	0.00e+00	7.35e-01	0.00e+00

Supplementary Table 1 Correlation among features for human miRNAs and mRNAs. Each cell contains the Spearman rank correlation coefficient (*r*) and the corresponding p-value (*p*).

		Trans.Grimson	Trans.Linsley	Prot.Selbach	IPcross.Chi	IP.Hendrickson	RibN.Hendrickson	Trans.Hendrickson
AU content	r	-0.229	-0.166	-0.163	0.160	0.249	-0.228	-0.293
AU content	p	1.17e-48	1.92e-57	1.70e-06	2.51e-05	8.02e-10	1.86e-08	3.47e-13
AU content	r′	-0.967	-0.868	-0.922	0.944	0.994	-0.982	-0.986
AU content	p′	4.68e-09	2.72e-05	8.97e-03	4.68e-03	5.13e-05	4.79e-04	2.87e-04
UTR position	r	0.117	0.060	0.064	-0.022	-0.278	0.125	0.206
UTR position	p	1.39e-13	8.33e-09	6.26e-02	5.63e-01	5.18e-12	2.20e-03	3.92e-07
UTR position	r′	0.880	0.643	0.612	-0.178	-0.921	0.774	0.804
UTR position	p′	1.51e-05	9.79e-03	1.96e-01	7.36e-01	9.02e-03	7.09e-02	5.36e-02
3′ pairing	r	-0.051	-0.041	-0.058	0.030	0.099	-0.043	-0.028
3′ pairing	p	1.16e-03	7.49e-05	9.13e-02	4.26e-01	1.62e-02	2.95e-01	4.90e-01
3′ pairing	r′	-0.493	-0.764	-0.757	0.167	0.607	-0.441	-0.269
3′ pairing	p′	6.20e-02	9.05e-04	8.17e-02	7.52e-01	2.02e-01	3.81e-01	6.06e-01
ΔG duplex	r	0.000	-0.062	-0.017	0.017	-0.002	-0.059	-0.055
ΔG duplex	p	9.87e-01	3.34e-09	6.13e-01	6.57e-01	9.53e-01	1.49e-01	1.79e-01
ΔG duplex	r′	0.039	-0.657	-0.167	-0.009	0.086	-0.521	-0.430
ΔG duplex	p′	8.89e-01	7.75e-03	7.52e-01	9.86e-01	8.71e-01	2.89e-01	3.94e-01
ΔG binding	r	0.023	-0.050	0.001	0.020	-0.007	-0.042	-0.027
ΔG binding	p	1.43e-01	1.70e-06	9.69e-01	5.99e-01	8.63e-01	3.11e-01	5.05e-01
ΔG binding	r′	0.320	-0.598	0.198	0.184	0.054	-0.563	-0.272
ΔG binding	p′	2.45e-01	1.85e-02	7.06e-01	7.27e-01	9.20e-01	2.44e-01	6.02e-01
ΔG open	r	0.196	0.107	0.085	-0.108	-0.234	0.194	0.257
ΔG open	p	4.42e-36	1.61e-24	1.31e-02	4.47e-03	7.40e-09	1.82e-06	1.95e-10
ΔG open	r′	0.950	0.788	0.939	-0.914	-0.875	0.949	0.947
ΔG open	p′	6.23e-08	4.79e-04	5.56e-03	1.09e-02	2.25e-02	3.84e-03	4.12e-03
ΔG total	r	0.204	0.086	0.078	-0.096	-0.249	0.183	0.251
ΔG total	p	8.31e-39	1.95e-16	2.28e-02	1.18e-02	7.02e-10	7.08e-06	5.09e-10
ΔG total	r′	0.939	0.833	0.735	-0.802	-0.824	0.865	0.940
ΔG total	p′	2.27e-07	1.17e-04	9.57e-02	5.51e-02	4.39e-02	2.60e-02	5.21e-03
P.over exact	r	0.170	0.065	0.073	0.015	-0.222	0.076	0.221
P.over exact	p	2.93e-27	4.12e-10	3.32e-02	7.02e-01	4.75e-08	6.45e-02	5.27e-08
P.over exact	r′	0.920	0.745	0.687	0.069	-0.875	0.914	0.837
P.over exact	p′	1.25e-06	1.45e-03	1.31e-01	8.97e-01	2.25e-02	1.07e-02	3.76e-02
P.over binomial	r	0.147	0.067	0.069	0.029	-0.207	0.085	0.188
P.over binomial	p	1.24e-20	1.08e-10	4.30e-02	4.40e-01	3.74e-07	3.84e-02	3.91e-06
P.over binomial	r′	0.867	0.634	0.702	0.329	-0.826	0.677	0.860
P.over binomial	p′	2.84e-05	1.12e-02	1.20e-01	5.24e-01	4.29e-02	1.40e-01	2.80e-02
BLS	r	-0.158	-0.108	-0.124	0.112	0.148	-0.160	-0.226
BLS	p	9.93e-24	2.99e-25	2.78e-04	3.36e-03	2.96e-04	8.95e-05	2.66e-08
BLS	r′	-0.954	-0.815	-0.931	0.893	0.661	-0.817	-0.883
BLS	p′	3.52e-08	2.14e-04	6.97e-03	1.66e-02	1.53e-01	4.70e-02	1.97e-02
PhyloP	r	-0.205	-0.143	-0.172	0.196	0.116	-0.155	-0.223
PhyloP	p	3.00e-39	2.97e-43	4.49e-07	2.22e-07	4.74e-03	1.49e-04	4.12e-08
PhyloP	r′	-0.949	-0.857	-0.946	0.856	0.719	-0.918	-0.929
PhyloP	p′	6.82e-08	4.46e-05	4.31e-03	2.96e-02	1.07e-01	9.83e-03	7.34e-03
PhastCons	r	-0.205	-0.128	-0.143	0.223	0.020	-0.093	-0.124
PhastCons	p	3.26e-39	6.51e-35	2.69e-05	3.22e-09	6.20e-01	2.35e-02	2.38e-03
PhastCons	r′	-0.920	-0.823	-0.975	0.905	0.157	-0.704	-0.690
PhastCons	p′	1.24e-06	1.67e-04	9.59e-04	1.31e-02	7.67e-01	1.18e-01	1.30e-01

Supplementary Table 2 Correlation between each feature and the seven experimental miRNA repression measures. Data points were binned in 15 equally-sized bins. r is the correlation on the full dataset (p: corresponding p-value); r' is the correlation on the binned dataset (p': corresponding p-value.

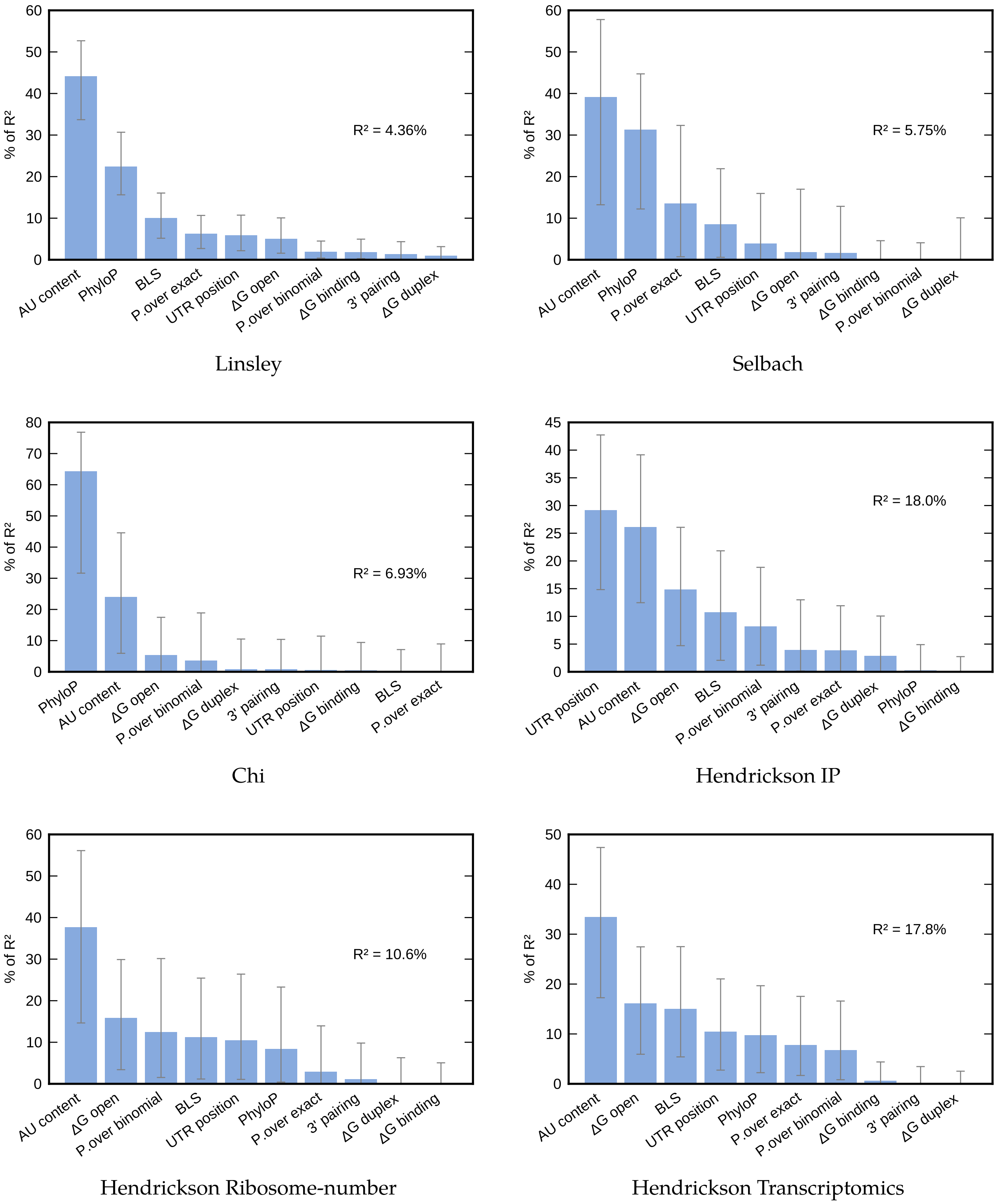

Supplementary Figure 1 Feature relative importance in the miRmap multiple linear regression models predicting miRNA repression strength for all datasets used in this study considering 7-mer seeds. R^2 is the proportion of variance explained by the model.

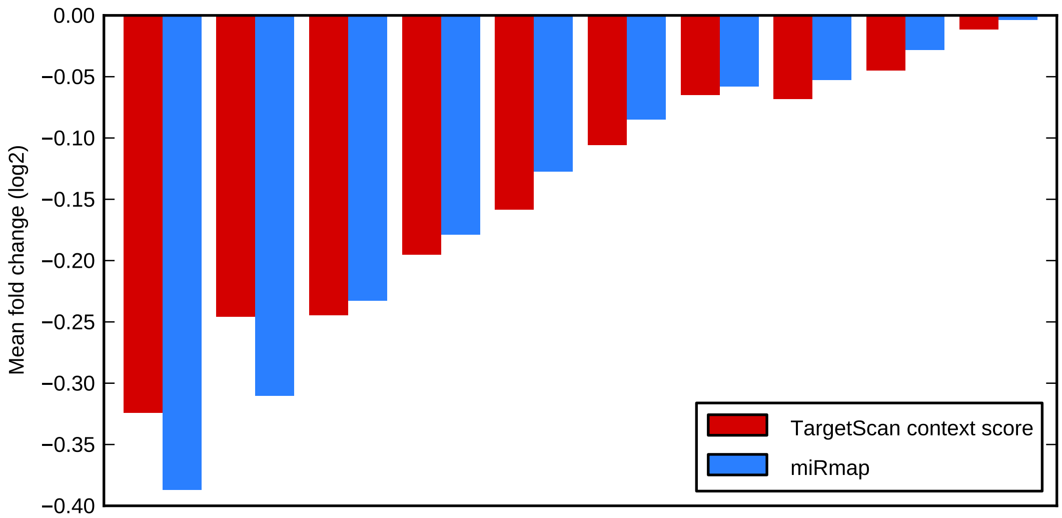

Supplementary Figure 2 Predictive performance of miRmap and TargetScan context score models for 7-mer seeds. All predicted miRNA-mRNA pairs were ranked according to their predicted strength and binned into 10 equally-sized bins. For each bin, the average experimental fold-change was computed. The same procedure was repeated with the miRmap and TargetScan context score models. (Trans.Grimson dataset)

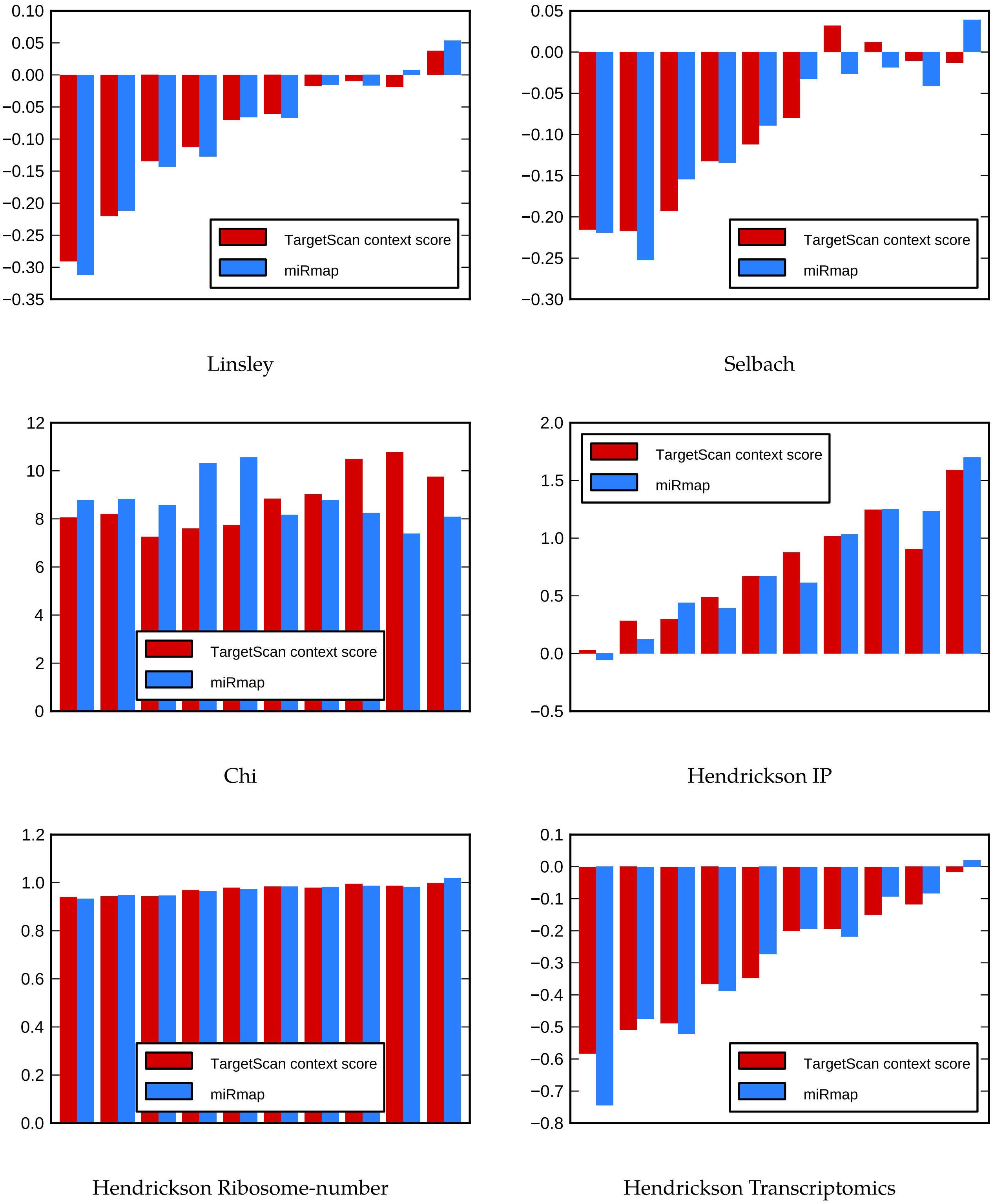

Supplementary Figure 3 Predictive performance of miRmap and TargetScan context score models for 7-mer seeds. All predicted miRNA-mRNA pairs were ranked according to their predicted strength and binned into 10 equally-sized bins. For each bin, the average experimental fold-change was computed. The same procedure was repeated with the miRmap and TargetScan context score models.

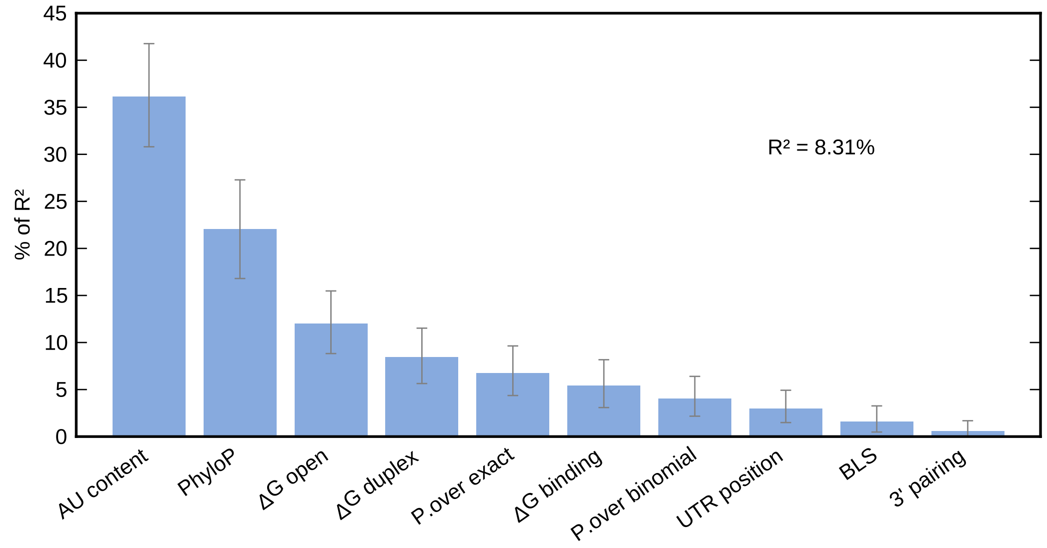

A

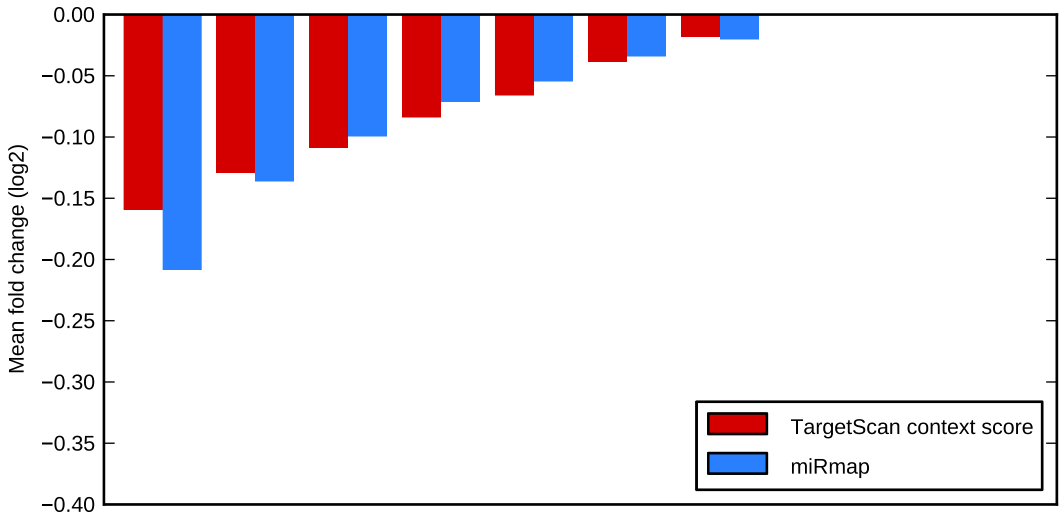

B

Supplementary Figure 4 Performance and feature relative importance for 6-mer seeds. The same procedures as in Figure 5B (A) and in Supplementary Figure 3 (B) were applied to the set of potential target sites defined by the presence of a single 6-mer seed-match. (Trans.Grimson dataset)

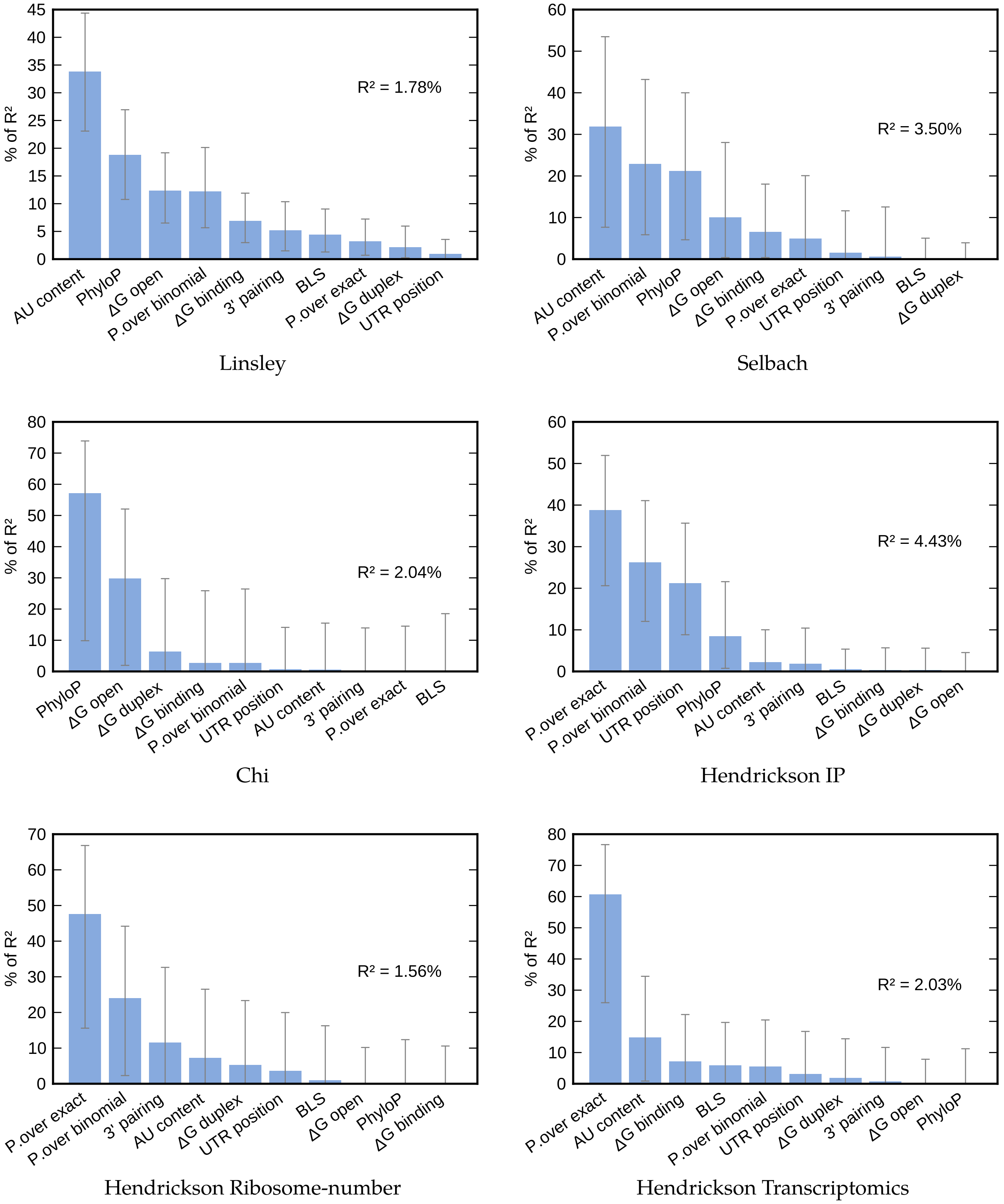

Supplementary Figure 5 Feature relative importance in the miRmap multiple linear regression models predicting miRNA repression strength for all datasets used in this study considering 6-mer seeds. R^2 is the proportion of variance explained by the model.

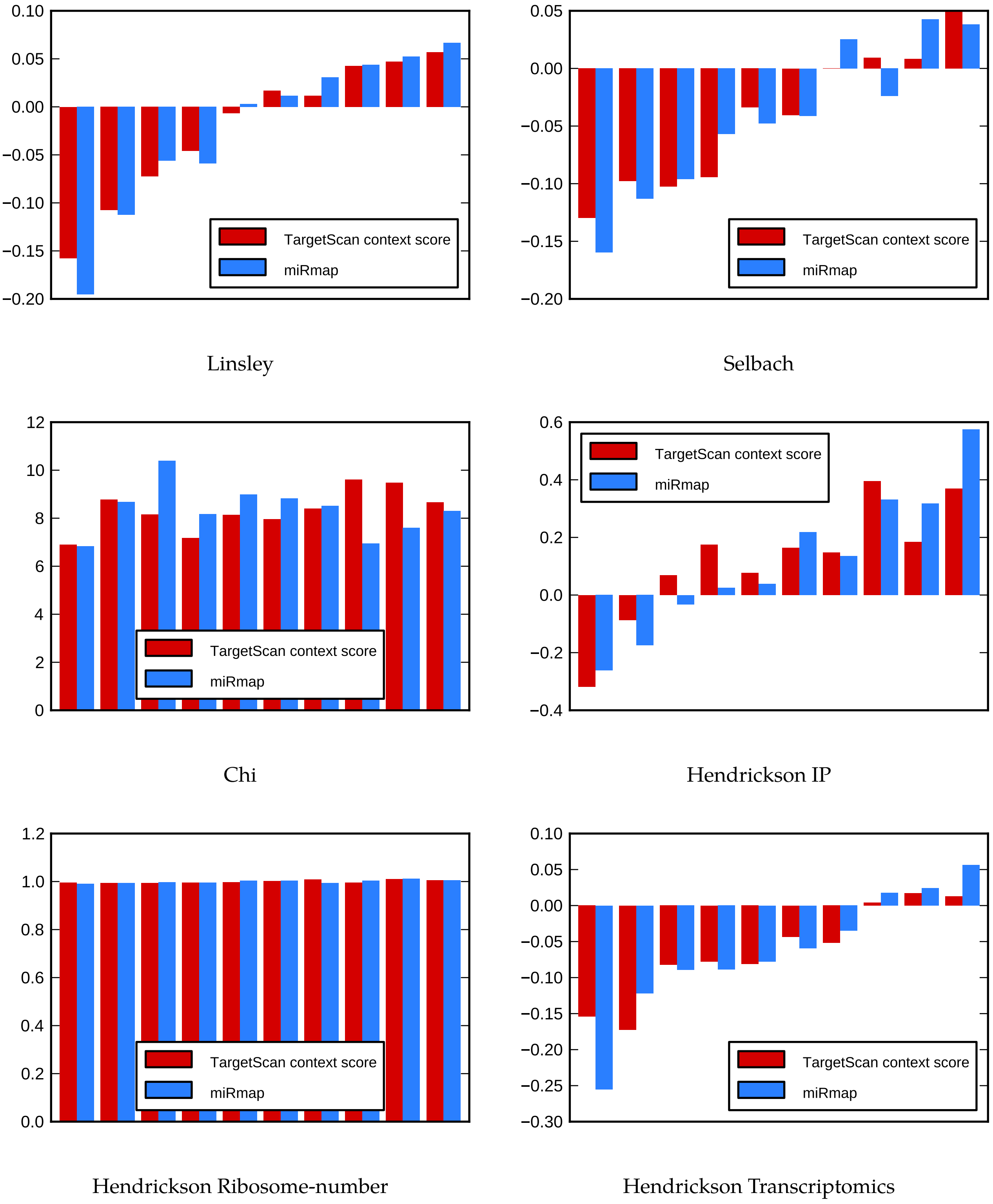

Supplementary Figure 6 Predictive performance of miRmap and TargetScan context score models for 6-mer seeds. All predicted miRNA-mRNA pairs were ranked according to their predicted strength and binned into 10 equally-sized bins. For each bin, the average experimental fold-change was computed. The same procedure was repeated with the miRmap and TargetScan context score models.

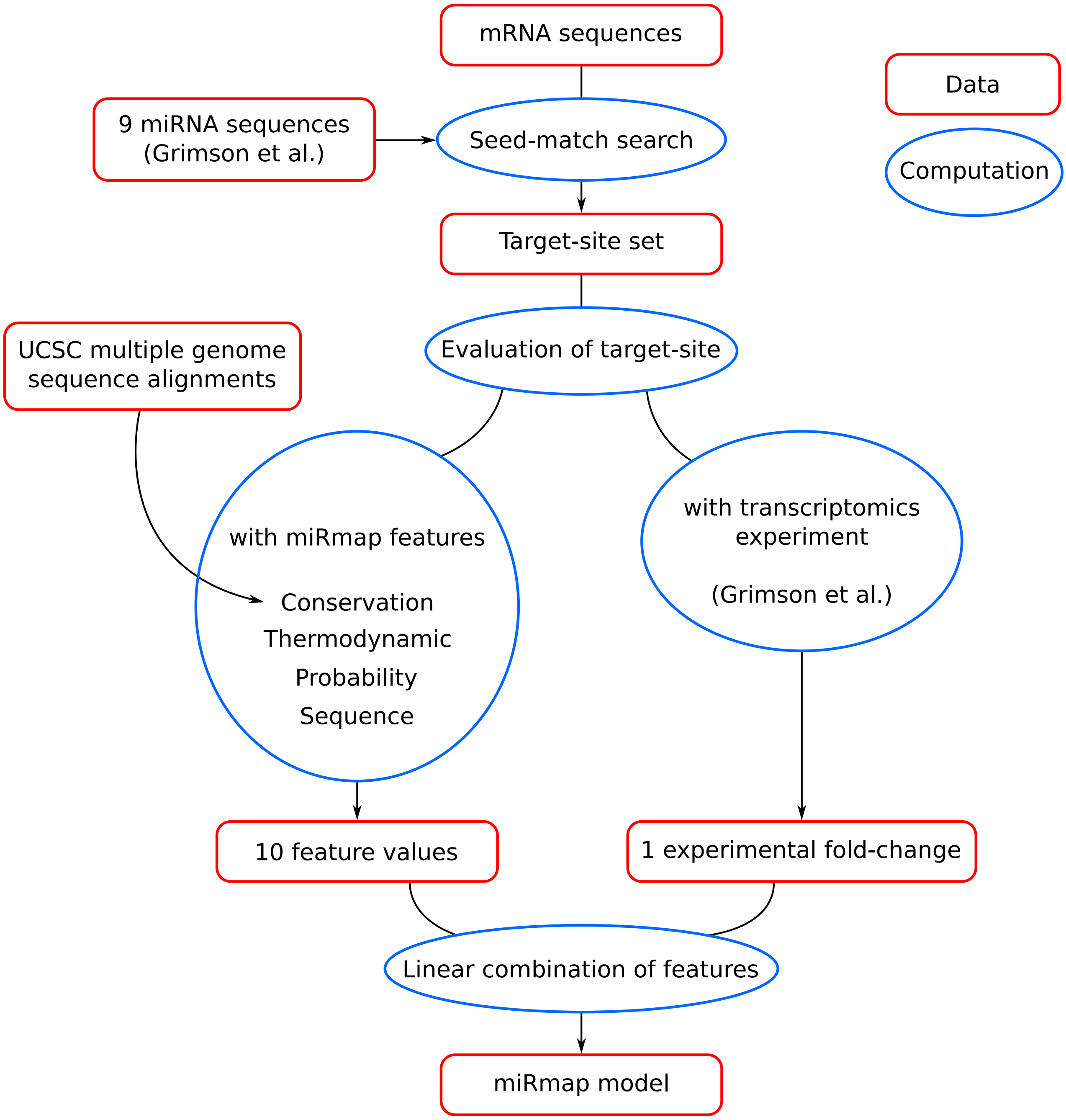

Supplementary Figure 7 Description of the miRmap model building. After the target site identification with seed-matches, their repression strength is evaluated computationally and experimentally. The linear combination of the features based on the experimental output is the miRmap model.

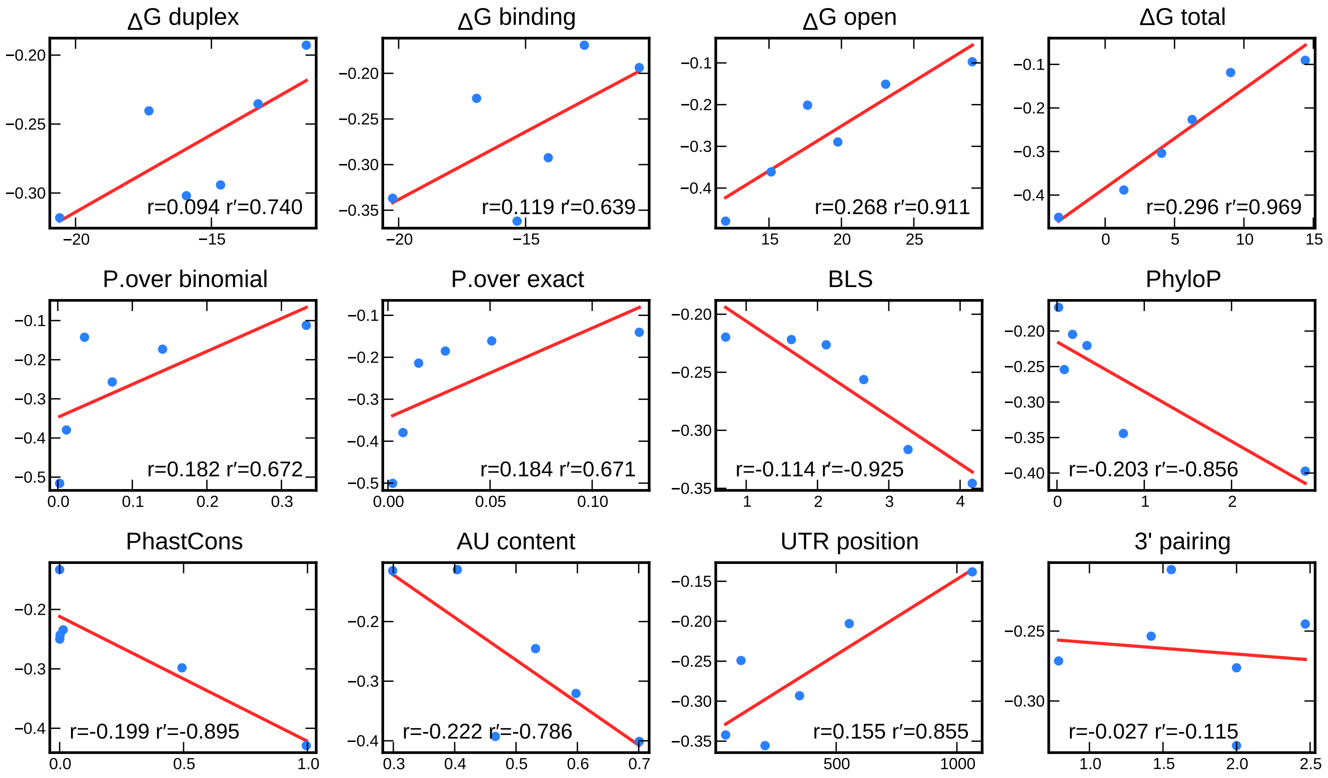

min()

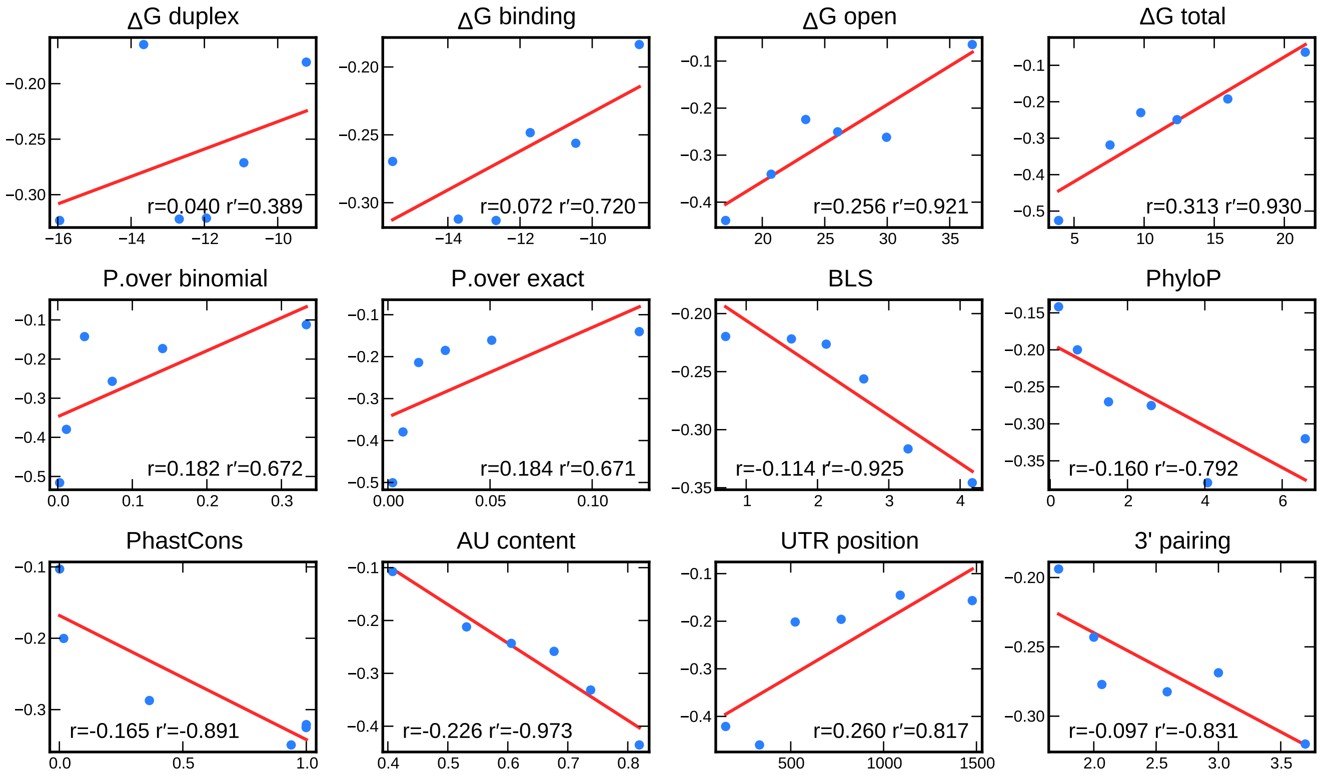

max()

Supplementary Figure 8 (see next page for legend)

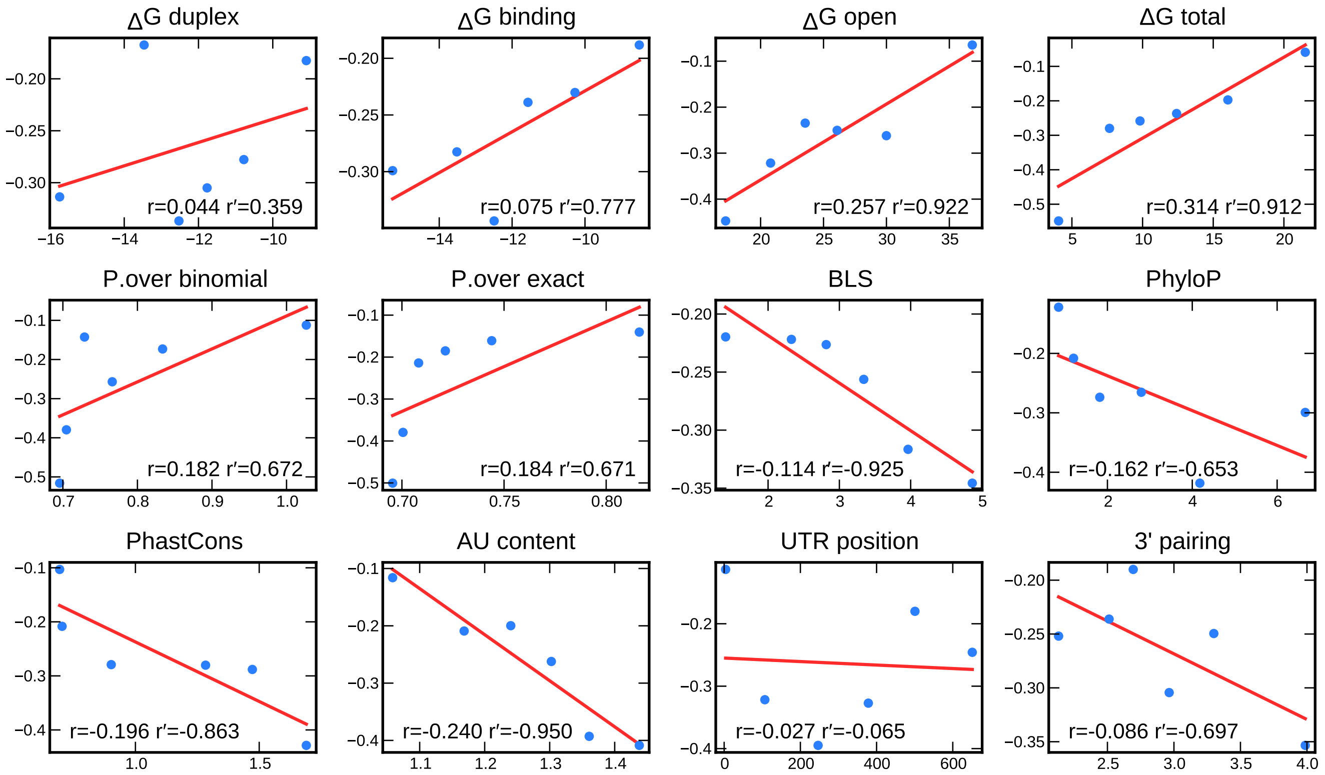

$\log(\sum exp())$

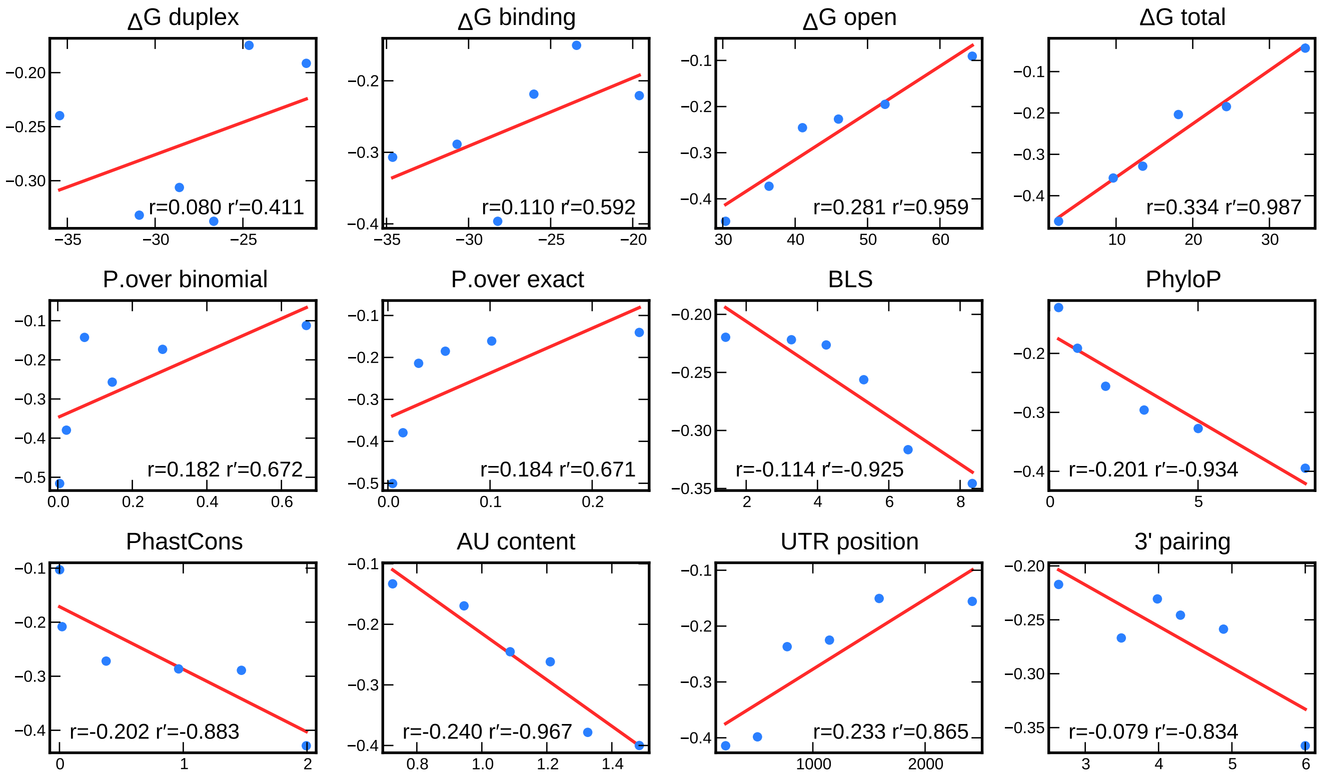

$\sum$

Supplementary Figure 8 Correlation similar to Figure 3 for mRNAs with 2 target sites (*Trans.Grimson* dataset) combined with 4 different functions, shown at the bottom of each picture block.
